# Oral Health Characteristics and Dental Rehabilitation of Children with Global Developmental Delay

**DOI:** 10.1155/2017/5486327

**Published:** 2017-01-30

**Authors:** Saurabh Kumar, Deepika Pai, Runki Saran

**Affiliations:** ^1^Department of Pedodontics & Preventive Dentistry, Manipal College of Dental Sciences, Manipal, India; ^2^Faculty of Dentistry, Melaka Manipal Medical College, Manipal, India

## Abstract

Global developmental delay (GDD) is a chronic neurological disturbance which includes defects in one or more developmental domains. The developmental domain can be motor, cognitive, daily activities, speech or language, and social or personal development. The etiology for GDD can be prenatal, perinatal, or postnatal. It can be diagnosed early in childhood as the delay or absence of one or more developmental milestones. Hence the role of pedodontist and pediatricians becomes more crucial in identifying this condition. The diagnosis of GDD requires a detailed history including family history and environmental risk factors followed by physical and neurological examinations. Investigations for GDD include diagnostic laboratory tests, brain imaging, and other evidence-based evaluations. GDD affects multiple developmental domains that not only have direct bearing on maintenance of oral health, but also require additional behavior management techniques to deliver optimal dental care. This paper describes two different spectra of children with GDD. Since the severity of GDD can vary, this paper also discusses the different behavior management techniques that were applied to provide dental treatment in such children.

## 1. Introduction

According to American Academy of Neurology (AAN) and Child Neurology Society (CNS), global developmental delay (GDD) is a subset of developmental disabilities defined as significant delay in two or more domains of development, including activities of daily living as well as motor, cognitive, speech/language, and personal/social skills [1, 2]. The development delay should be evident in comparison with attainment of skills to their chronological peers. Those deficits are evident in comparison with the skills attainment of chronological peers. Significant delay is defined as performance of two standard deviations or more below the mean on age-appropriate, standardized norm referenced testing [3, 4]. The prevalence of GDD is not precisely estimated [5]. The etiology can be intrapartum asphyxia, cerebral dysgenesis, early severe psychosocial deprivation, antenatal toxin exposure like alcoholism or multidrug exposures, chromosomal disorders including Down syndrome, fragile X syndrome, and Rett's syndrome [6, 7].

Diagnosis of GDD is comprehensive one which must include a detailed history, thorough clinical examination, and appropriate investigations. Most often a diagnosis can be made based on detailed history and clinical evaluation. A thorough prenatal, natal, and postnatal history along with family history is essential and is the first step towards establishing the diagnosis. Clinical examination for GDD includes neuromuscular examination and examination of the spine, reflexes, gait, and vision. In order to diagnose a case of GDD the investigations can be performed selectively or guided by history and clinical features. Most often a clinical diagnosis is sufficient. Hence if required the pediatrician can perform metabolic investigations, neuroimaging, or advanced genetic investigations [8].

Clinical presentation of GDD may not be uniform, as it depends on the domains affected. Clinical features may include one or more of the following dysmorphic features like short stature, macrocephaly, generalized hair growth anomalies, facial asymmetry, flat facial profile, and midface hypoplasia. Epilepsy is a common finding with children having GDD. Oral findings include drug induced gingival hyperplasia if the child is on medication for epilepsy [7]. Due to motor and cognitive developmental delay, children with GDD also display poor oral hygiene and dental caries commensurate to inadequate plaque control. We hereby present two cases of varying intensities of GDD, their oral findings, and behavior management techniques which were applied for dental treatment.

## 2. Case Reports

### 2.1. Case 1

**Figure 1:**
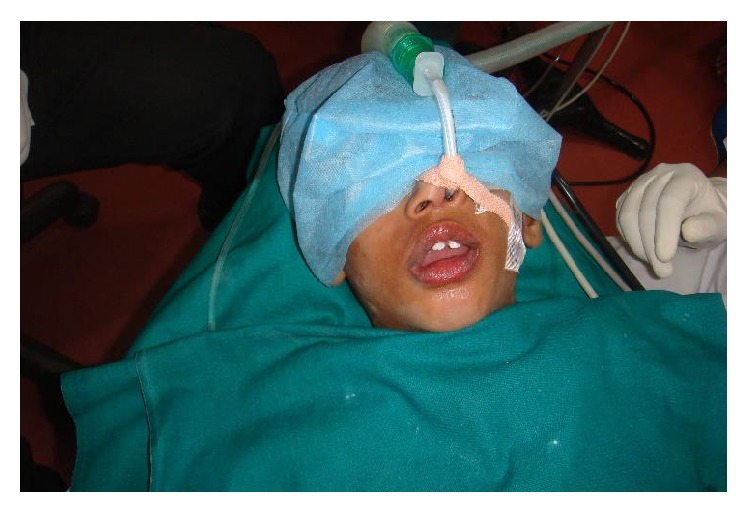
Dental treatment under general anesthesia (Case 1).

**Figure 2:**
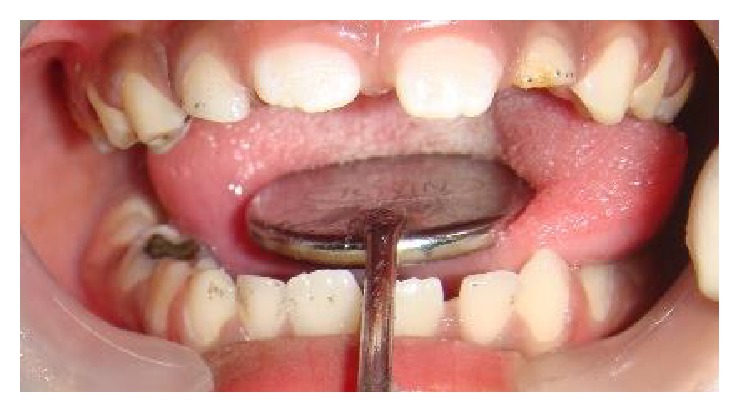
Preoperative photograph (Case 1).

**Figure 3:**
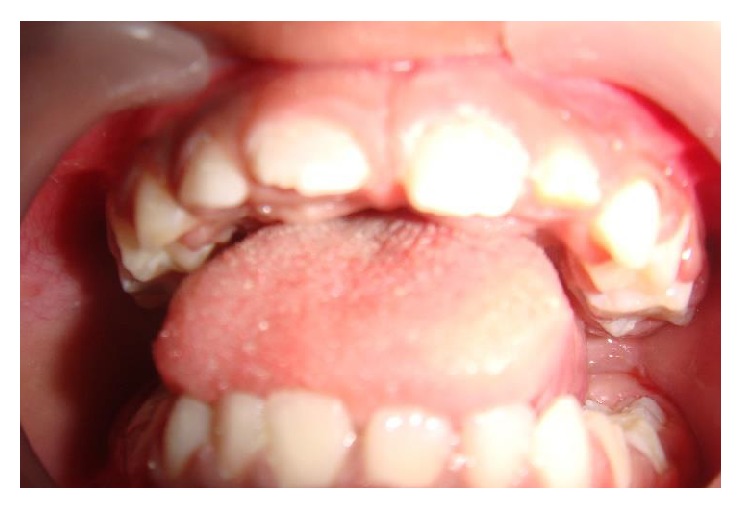
Postoperative photograph (Case 1).

A seven-year-old boy reported to our clinic with the chief complaint of multiple decayed teeth. The child was a known case of global developmental delay with seizure disorder. There was no familial history of any neurological problem. Due to the developmental delay in speech, language, and cognition, a thorough oral examination was not possible. The child's behavior was categorized as definitely negative according to Frankl's behavior rating scale. Since the child's ability to cooperate was limited due to presence of GDD additionally the child was a known epileptic, the dental treatment was planned under chairside general anesthesia. After obtaining necessary consents and clearance from the department of pediatrics and anesthesiology the child was scheduled for dental rehabilitation under chairside general anesthesia (Figure 1). Oral examination under general anesthesia revealed mixed dentition with multiple decayed teeth and poor oral hygiene (Figure 2). The dental treatment including oral prophylaxis, pit and fissure sealant (Clinpro Sealant, 3M ESPE) application on all the permanent first molars, the Glass ionomer cement (Fuji IX) restorations of the carious primary second molars, and bifluoride varnish (VOCO) application was done under general anesthesia (Figure 3).

### 2.2. Case 2

**Figure 4:**
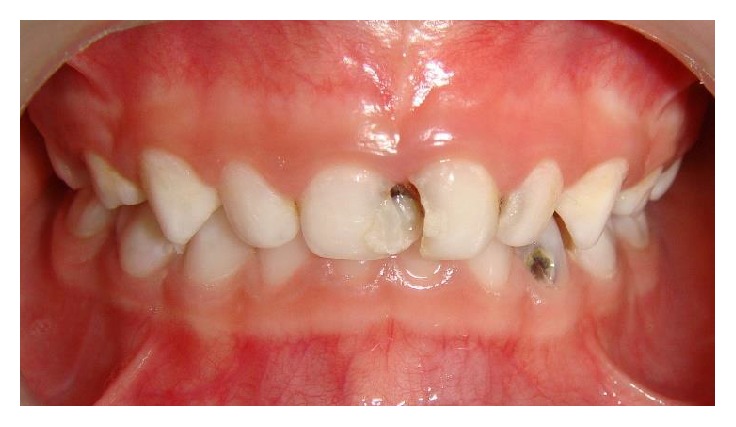
Preoperative photograph (Case 2).

**Figure 5:**
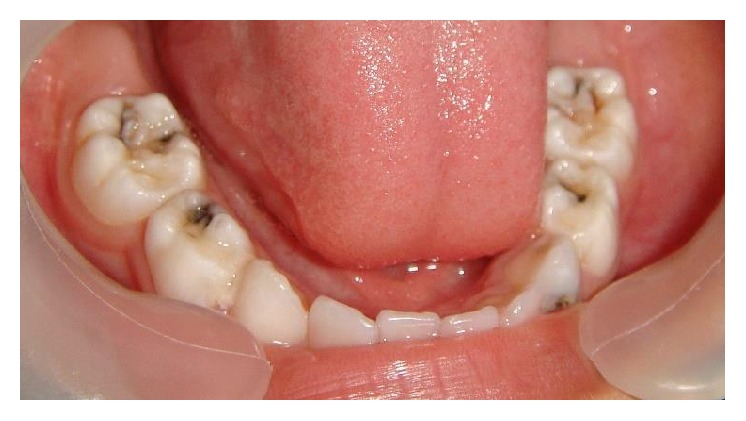
Preoperative photograph (Case 2).

**Figure 6:**
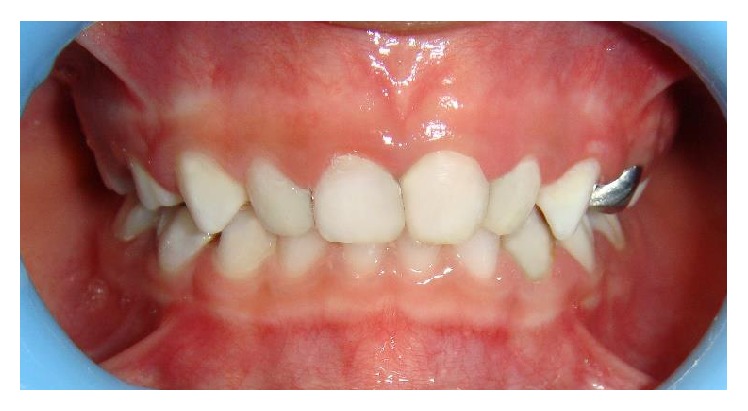
Postoperative photograph (Case 2).

**Figure 7:**
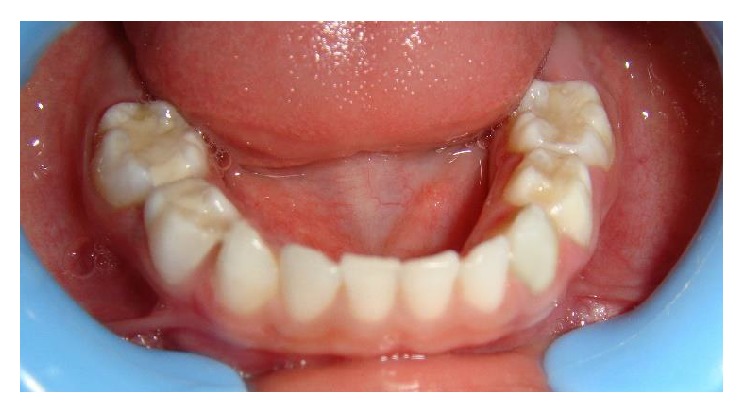
Postoperative photograph (Case 2).

A six-year-old male child was referred by his pediatrician for dental treatment of decayed teeth. He was a known case of global developmental delay since the age of two years as his parents found that his milestones like walking and speech were delayed. Mother gave the history of her son who had difficulty in learning at school. He was attending special school and also was being rehabilitated at center for children with neuromuscular disorders. Upon review with his pediatrician we learnt that he had a mild degree of developmental delay and predominance in the development of speech and communication but was rehabilitated enough to vocalize in English fairly well. Frankl's behavior rating for this child was positive. On examination the carious lesions were present on the primary molars and maxillary incisors (Figures 4 and 5). Oral prophylaxis was accomplished with behavior management and physical restraining. However owing to uncontrolled tongue movement and instable jaw movements the restorations including stainless steel crown placement were done under oral sedation with Pedicloryl (dosage: 0.5–1 mg/kg) (Figures 6 and 7). APF gel was applied as a preventive protocol. The patient was advised to use powered toothbrushes for maintaining oral hygiene. He was reviewed periodically at every 3 months interval.

## 3. Discussion

As the clinical presentation of GDD may be heterogeneous the oral findings depend on the developmental domains affected. Hence both the treatment needs and behavior management techniques depend on individual case presentation. Developmental domains such as gross/fine motor skills, speech and language, cognitive development, social/personal development, and activities of daily living are affected by GDD [1].

Functional motor disability can lead to uncontrolled movements of head and neck and also involuntary movements of tongue that can interfere in delivering optimal dental treatment. Excessive drooling of saliva and poor manual dexterity due to motor disability can also influence the effectiveness of tooth brushing. Occupational therapy towards neuromuscular coordination and training can improve the motor disability over time [9]. Delay or deficiencies in development of speech, language, and cognition definitely act as a hindrance to appropriate delivery of oral care. If social/personal development is affected, the child may not be attending school or may not want to visit a dentist too. The pedodontist should build a good rapport with such children so as to be familiar with the child. Tooth brushing is a routine daily activity; hence if the domain of daily activities remains affected in a child with GDD, they are definitely vulnerable for poor oral hygiene and so it increases their risk for development of dental caries. In such situations powered toothbrushes act as an excellent method for achieving satisfactory plaque control [10].

A universal behavior management protocol cannot be adopted for children with GDD. As in our cases, Case 1 was an extreme case of GDD with epilepsy with developmental delay in multiple domains. Hence general anesthesia was considered the best management option for complete dental rehabilitation. Unlike the first case the child in Case 2 was a mild case of GDD, had no other underlying systemic illness detected, and also was adequately rehabilitated at neurology center. This facilitated chair side oral examination and minimally invasive treatments like oral prophylaxis. Since salivary contamination and unstable head and neck movements would compromise the quality of the restoration, the restorations of decayed teeth were done under oral sedation.

The objective of treatment planning in cases with GDD therefore should encompass the assessment of level of cooperating ability for delivering oral care, a thorough review of the underlying medical conditions, and possible drug therapy for the systemic conditions along with oral health status of the individual.

## 4. Conclusion

Since pediatricians and pedodontist are the first of the health care providers to examine a child for developmental milestones. A careful history and examination can lead to early detection of GDD. Adequate rehabilitation of these children at occupational therapy centers can also enable them to improve the quality of life at large along with their oral health. Hence we emphasize the role of pedodontist in early detection of GDD and helping these children gain better oral and general health.

## References

[1] Shevell M., Ashwal S., Donley D. (2003). Practice parameter: evaluation of the child with global developmental delay: report of the quality standards subcommittee of the American Academy of Neurology and The Practice Committee of the Child Neurology Society. *Neurology*.

[2] AAN Guideline Summary for clinicians, http://www.childneurologysociety.org/

[3] Majnemer A., Shevell M. I. (1995). Diagnostic yield of the neurologic assessment of the developmentally delayed child. *The Journal of Pediatrics*.

[4] Shevell M. I., Majnemer A., Rosenbaum P., Abrahamowicz M. (2000). Etiologic yield of subspecialists' evaluation of young children with global developmental delay. *Journal of Pediatrics*.

[5] Yeargin-Allsopp M., Murphy C. C., Cordero J. F., Decouflé P., Hollowell J. G. (1997). Reported biomedical causes and associated medical conditions for mental retardation among 10 year old children, metropolitan Atlanta, 1985 to 1987. *Developmental Medicine and Child Neurology*.

[6] Shevell M. (2008). Global developmental delay and mental retardation or intellectual disability: conceptualization, evaluation, and etiology. *Pediatric Clinics of North America*.

[7] Patil R. B., Urs P., Kiran S., Bargale S. D. (2014). Global developmental delay with sodium valproate-induced gingival hyperplasia. *BMJ Case Reports*.

[8] McDonald L., Rennie A., Tolmie J., Galloway P., McWilliam R. (2006). Investigation of global developmental delay. *Archives of Disease in Childhood*.

[9] Sorsdahl A. B., Moe-Nilssen R., Kaale H. K., Rieber J., Strand L. I. (2010). Change in basic motor abilities, quality of movement and everyday activities following intensive, goal-directed, activity-focused physiotherapy in a group setting for children with cerebral palsy. *BMC Pediatrics*.

[10] Doğan M. C., Alaçam A., Aşici N., Odabaş M., Seydaoğlu G. (2009). Clinical evaluation of the plaque‐removing ability of three different toothbrushes in a mentally disabled group. *Acta Odontologica Scandinavica*.

